# Estimating Mode of Transport in Daily Mobility during the COVID-19 Pandemic Using a Multinomial Logistic Regression Model

**DOI:** 10.3390/ijerph20054600

**Published:** 2023-03-05

**Authors:** Jaroslav Mazanec, Veronika Harantová, Vladimíra Štefancová, Hana Brůhová Foltýnová

**Affiliations:** 1 Department of Quantitative Methods and Economic Informatics, Faculty of Operation and Economics of Transport and Communications, University of Zilina, 01026 Zilina, Slovakia; 2 Department of Road and Urban Transport, Faculty of Operation and Economics of Transport and Communications, University of Zilina, 01026 Zilina, Slovakia; 3 Department of Railway Transport, Faculty of Operation and Economics of Transport and Communications, University of Zilina, 01026 Zilina, Slovakia; 4 Faculty of Social and Economic Studies, Jan Evangelista Purkyňe University in Ústí nad Labem, 40096 Ústí nad Labem, Czech Republic

**Keywords:** COVID-19, mobility, multinomial regression model, transport

## Abstract

At the beginning of 2020 there was a spinning point in the travel behavior of people around the world because of the pandemic and its consequences. This paper analyzes the specific behavior of travelers commuting to work or school during the COVID-19 pandemic based on a sample of 2000 respondents from two countries. We obtained data from an online survey, applying multinomial regression analysis. The results demonstrate the multinomial model with an accuracy of almost 70% that estimates the most used modes of transport (walking, public transport, car) based on independent variables. The respondents preferred the car as the most frequently used means of transport. However, commuters without car prefer public transport to walking. This prediction model could be a tool for planning and creating transport policy, especially in exceptional cases such as the limitation of public transport activities. Therefore, predicting travel behavior is essential for policymaking based on people’s travel needs.

## 1. Introduction

The COVID-19 pandemic has changed the world and affected most countries and cities worldwide, affecting how people live and work. Many countries around the world have taken extraordinary measures to avoid social contact to prevent the further spread of the virus [1,2]. Isolation and quarantine measures were successful in reducing the earlier spread of SARS [3]. In the beginning, mobility restrictions were introduced for all modes of transport [4]. Later, other measures were introduced, such as socio-economic restrictions, physical distancing, and hygiene measures [5]. Lockdown measures have significantly affected travel behaviour, especially people’s mode of transport [6]. These measures were necessary to prevent the transmission of COVID-19, which was a problem for people who must leave for work or buy basic food. The best way to prevent and slow down the transmission of COVID-19 is to follow three basic rules: cover your nose and mouth with a mask, wash and disinfect your hands, and keep safe distances to limit the spread [7]. From the available literature, we are unaware of any research focusing on the factors influencing travel mode choice of a sample of people from two countries. Our research aimed to find statistically significant factors affecting the choice of means of transport and to develop a model based on independent variables such as economic status, residence, and ownership of a car in the household, to assess the most common means of transport such as car, bus, public transport, and walking. We focused on travel habits, and behavior during the pandemic obtained using an anonymous online questionnaire. All these inputs, such as economic status, residence, and car ownership in the household were statistically significant variables estimating the dependent variable. We focused on the travel habits of two states that once formed a single state, as travelers represent a sample with similar travel habits. The purpose of the paper was to attract selected commuters to work or school to use public transport or alternative means of transport, such as shared scooters or bicycles, to reduce carbon footprint and eliminate traffic peaks in the morning and afternoon hours. The results contribute to planning and building a public transport or integrated transport policy not only at the national but also at the international level. This understanding is important, and we consider the sample to be specific. Predicting travel behavior is imperative for transportation planning, decision-making, and policymaking during a pandemic, based on people’s travel needs.

The paper consists of an introduction, literature review, and methodology including the sample description and methods used. The following part shows the results of modelling mobility. Finally, the paper presents a discussion and our conclusions.

## 2. Literature Review

Several studies have used human mobility data to analyze emerging travel activities and travel behavior in Asia and Europe during the COVID-19 pandemic [8,9,10,11,12]. The results show that the strict control measures implemented by the government mitigated the spread of COVID-19 and significantly reduced the proportion of travel activities. There are also more detailed studies of mobility based on regression models for countries such as Hungary [13], Italy [14] New Zealand [15] that associate travel behavior with socio-economic variables and restrictions on mobility. Statistics show a significant reduction in public transport demand also in countries with relatively weak COVID-19 pandemic effects, such as New Zealand. Socio-economic activities have changed, and many people work from home or are unemployed, and mobility habits and patterns changed during and after the lockdown period. These changes in the choice of transport may take a long time to change after the COVID-19 pandemic [16].

Belik et al. showed that repetitive daily commuting and social activities led to different rates of spread of the pandemic [17]. From the point of view of gender difference, decisions about the choice of transport differ between men and women. Women are more likely than men to use public and active transport modes [18]. Pinchoff et al. conducted a household survey in five urban slums to describe factors associated with mobility over a 24 h period. In adjusted multinomial regression models, women were more than 58 % more likely than men to stay home during the pandemic [19].

At the same time, the pandemic revealed the fear of using common spaces, such as public transport, where it is difficult to observe social distancing, and the significantly increased of spreading the infection. Studies also describe the operation of public transport modes without restrictions during a pandemic, provided that its users observe preventive measures [20]. A UK survey found that 72% of respondents would not use public transport unless safety and hygiene measures were in place, while 18% of respondents were happy to resume services as soon as government restrictions were lifted [21]. Of course, the loyalty of certain groups on public transport can also be affected by purchase of subscription tickets. Jenelius found that passengers in Sweden switched from monthly season tickets to single tickets, while the use and sales of short-term season tickets dropped to almost zero. The number of one-year travel tickets and school tickets increased, showing that passengers using these tickets were tied to the public transport system [22]. A deep understanding of subscriber behavior is vital not only to improve services but also to maximize user retention, as Wang stated in his research [23]. During the pandemic, however, it is necessary to create a safe environment for traveling by public transport and find key factors. In practice, these factors cannot be considered in isolation from each other or the many other direct and indirect influences on the demand for public transport.

Paulley et al. [24] claim that income and vehicle ownership are the key factors having a major impact on travel and the choice of mode of transport. Another factor affecting the choice of means of transport is the place of residence; people living in the city have a higher tendency to use public transport (especially in cities with a metro) [25,26] unlike people living in the countryside [27]. Due to better and faster accessibility, people living outside the city use a car more often, and the pandemic forced city dwellers to switch to cars [28]. Several users retreated to their private cars and left the public space for fear of contagion [29,30]. A modal shift was also described, according to which 5% of respondents in India switched from public to private transport according to Pawar et al. [31]. Bucsky [13] reported that vehicle use increased from 43% to 65% in Budapest during the pandemic. Haas et al. [32] found that people in the Netherlands preferred to use cars and avoid public transport due to the coronavirus crisis. In Australia, a private vehicle was considered the most convenient mode of transport during the pandemic [33]. Due to the deteriorating economic situation, traveling by car was not a long-term preferred possibility, due to its costly nature. People could also choose other modes, such as walking and cycling. Many cities around the world hit hard by the pandemic saw a decrease in the number of cars on the road and an increase in cycling and walking trips.

These modes of transport increased during the pandemic, and were the most used in terms of social distancing [34,35,36,37]. The authors of [38] conducted a factor analysis to examine the underlying factors influencing mode choice before and during the pandemic. The results showed that the use of public transport fell, while walking and cycling increased slightly, during the pandemic. Respondents placed more emphasis on safety and security, comfort, cleanliness, fear of infection, personal social status, and availability of hand sanitizers. Teixeira and Lopes [39] observed users in a bike-sharing system in New York City during an outbreak. The bike share system had a smaller drop in ridership than public transport and an increase in the average journey length of 6 min. Using multinomial logistic regression, Costa [40] analyzed cyclists’ preferences in Lisbon during the pandemic. The results showed that people tended to cycle more often after the lockdown than before, two out of five cyclists cycled more often, on other hand two out of five supported more cycling frequency. The authors of the study from Bangladesh found that 45% of respondents were expected to increase bicycle travel, or during the new normal situation [41].

Disruptive events can lead to the discovery of facultative changes in people’s mobility and habits, and the pandemic could result in a more sustainable urban transport system [42]. 

Therefore, we decided to assess changes in travel behavior on a random sample using multinomial regression, as described in the next section.

## 3. Methodology

The methodology included a questionnaire for collecting data and methods for analysing travel behavior during the COVID-19 pandemic. We assessed the impact of various variables using a multinomial logistic model. The following hypotheses were formulated for our research:

**H1.**  *There is a statistically significant relationship between the economic status and transport modes for commuting to work or school.*

**H2.**  *There is a statistically significant relationship between the member household with the car and transport modes for commuting to work or school.*

**H3.**  *There is a statistically significant relationship between residence and transport modes for commuting to work or school.*

We aimed to find statistically significant factors affecting the choice of means of transport when commuting to work or school during the COVID-19 pandemic in the Czech Republic and Slovakia using multinomial logistic regression analysis.

### 3.1. Mobility Changes

Many studies based research on changes in travel behavior to COVID-19 on daily mobility reports from Google companies [43,44,45]. Google’s COVID-19 community mobility reports [46] show a clear decline in mobility patterns during the period of mandatory restrictions in the Czech Republic and Slovakia. The state of emergency began on 12 March 2020 in both countries.

**Figure 1:**
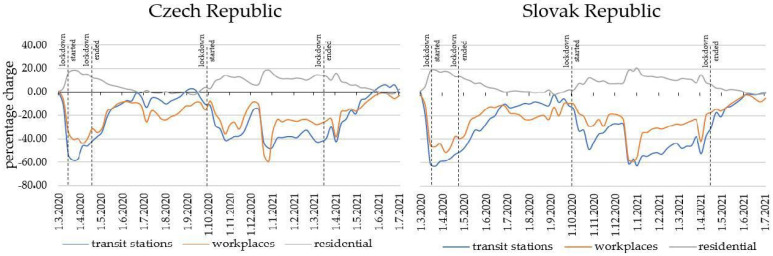
Google’s COVID-19 community mobility report for Czech and Slovak Republic.

Figure 1 shows the decline in mobility trends in different areas. The decline in mobility patterns started about a week before the lockdown was adopted in both countries, and peaked when mobility values approached zero. The decline in mobility began about a week before the adoption of the lockdown in both countries, ranging from −40% for workplaces to −60% at transport stations. On the other hand, mobility in residence surroundings increased by up to 20%. Among the factors that caused such fluctuations were mainly the home office, the closing of selected types of shops, and the cancellation of public gatherings. As the lockdown scenarios were repeated, we decided to conduct a mobility survey after the lockdown in 2021 to obtain mobility data during the pandemic period.

### 3.2. Data

First, we identify the minimum sample size of respondents for both countries. The Slovak Republic has almost 5.5 million inhabitants, and the Czech Republic has more than 10.5 million. Based on the formula for calculating the minimum sample size, we found that the sample should have at least 385 respondents.

(1) $$\mathrm{minimum}\;\mathrm{number}=\frac{\frac{z^{2}\;\text{×}\;p\;\times \;\left(1-\;p\right)}{e^{2}}}{1+\frac{z^{2}\;\times \;p\;\times \;\left(1\;-\;p\right)}{e^{2}\times \;n}}$$

(2) $$385=\frac{\frac{1.96^{2}\;\times \;0.5\;\times \;\left(1-\;0.5\right)}{0.05^{2}}}{1+\frac{1.96^{2}\;\times \;0.5\;\times \;\left(1-\;0.5\right)}{0.05^{2}\times \;16,228,257}}$$

where:

n: Total sample,
z: Critical minimal value for 95% confidence level is equal to 1.96,
p: The estimated proportion of the population that has the attribute in question (0.5 is recommended for unknown values),
e: Margin of error is equal to 0.05.

Our research was based on a total sample consisting of almost 2000 respondents from the Czech and Slovak Republics. However, the final sample consisted of almost 1500 respondents, as other respondents were excluded due to missing data describing passenger behavior and mobility during the COVID-19 pandemic. As can be seen, almost 70% of respondents were students, and the others were employees. These respondents were divided into eight subgroups based on input data. Table 1 shows the input variables for determining the means of transport for commuting to work or school based on independent variables such as economic status, residence, and car ownership in the household, using multinomial logistic regression analysis. All these input variables are categorical (nominal) variables. We found that the respondents most often commuted to work or school by car (more than 50%). In addition, respondents walked (almost 25%) or used public transport (less than 20%) to work or school. The sample was random. Our research did not require written informed consent from participants. All respondents expressed their consent through their voluntary participation in the research, the questionnaire did not have any personal data, and the questionnaire was anonymous. The online questionnaire was available from May to June 2021. During the pandemic, an online survey was a relevant tool for obtaining information about travel behavior and other passenger preferences [47]. The advantage was mainly in the speed of data acquisition, but also the safety of respondents during the COVID-19 pandemic. The respondents could express their opinion from the comfort of their homes [48]. The main goal of the questionnaire was to capture the travel behavior of travelers as a result of the COVID-19 lockdown. We designed a survey to increase the chance of obtaining the maximum number of responses, as a demanding survey could discourage respondents. We divided the survey into two parts. The first part concerned demographic data such as age, gender, economic status, and place of residence. The second part was focused on methods of travel, modes of transport, and availability of a car.

### 3.3. Methods

Multinomial logistic regression was used to predict categorical variables or the probability of belonging to a category for a response (dependent) variable based on multiple independent variables. As with binary logistic regression, multinomial logistic regression uses maximum likelihood estimation to estimate the probability of categorical membership. Thus, this type of model allowed us to characterize the probability of a respondent’s decision for a given multinomial discrete choice, depending on the values of the explanatory variables [49].

Multinomial logistic regression consists of several steps, such as assessing the explained variability based on Cox & Snell, Nagelkerke, and McFadden, and evaluating the significance of the overall model based on the Chi-Square Test. Model fitting information is another way to evaluate the model, compare the significance of the only-intercept model and the model with predictors, determine the significance of each independent variable using Likelihood Ratio Tests, determine the coefficients in multinomial regression, and estimate the probability of the response variable based on a unit change in the independent variable, others predictors being constant [50].

Assumptions. The response variable is a nominal variable with three or more categories. Categories of the outcome variables must be mutually exclusive and exhaustive. Independent variables are one or more independent continuous, ordinal, or nominal variables. The method requires independence of observations, no multicollinearity between independent variables, a linear relationship between any continuous independent variable, logit transformation of the response variable, and no outlier [50,51]. Multinomial logistic regression is expressed as

(3) $$P\left[Y=j/x\right]=\pi_{j}\left(x\right)=\frac{exp\left[g_{j}\left(x\right)\right]}{\sum_{k=0}^{r-1}\left[g_{k}\left(x\right)\right]}=\frac{\exp\left(\beta_{j0}+\beta_{j1}x_{2}+\beta_{j2}x_{2}+\cdots +\beta_{jm}x_{m}\right)}{\sum_{k=0}^{r-1}\exp\left(\beta_{k0}++\beta_{k1}x_{2}+\beta_{k2}x_{2}+\cdots +\beta_{kp}x_{p}\right)}$$

where:

$P\left[Y=j/x\right]$: conditional probability from response variable Y for the j category on the vector *x*, *j* = 0, 1, …, r − 1,
$\pi_{j}\left(x\right):$ the logistic regression model for response variable Y for the *j* category,
$g_{j}\left(x\right):$ the model logit for the j category,
$g_{j}\left(x\right):$ the model logit for the k category,
*X_k_*: the value of the m explanatory, *m* = 1, 2, 3, … *p*,
$\beta_{jm}:$ the coefficient parameter estimators, where vector ${\tilde{\beta }}_{0}=0$, $g_{0}\left(x\right)=0$,
${\tilde{\beta }}_{0}:$ parameter coefficient of the logit model or the response variable Y to the 0 category $\beta_{00},\;\beta_{01,}\cdots ,\;\beta_{0p}$ [52,53].

Log Likelihood Function (−2 LL) is a statistical measure, like total sums of squares in the regression model. If the measure decreases, this shows that there is a relationship between the response and independent variables. The function of conditional Likelihood for the sample of as many as n observations is written as:

(4) $$l\left(\beta \right)=\overset{n}{\underset{i=1}{\prod }}\left[\pi_{0}{\left(x_{i}\right)}^{y_{0i}}\pi_{1}{\left(x_{i}\right)}^{y_{1i}}\pi_{2}{\left(x_{i}\right)}^{y_{2i}}\;\cdots {\;\pi }_{r-1}{\left(x_{i}\right)}^{y_{\left(r-1\right)i}}\right]$$

If natural algorithms are taken of both sides, the Log-Likelihood function is expressed as:

(5) $$L\left(\beta \right)=\ln\left[l\left(\beta \right)\right]$$

(6) $$L\left(\beta \right)=\ln\left[\overset{n}{\underset{i=1}{\prod }}\left[\pi_{0}{\left(x_{i}\right)}^{y_{0i}}\pi_{1}{\left(x_{i}\right)}^{y_{1i}}\pi_{2}{\left(x_{i}\right)}^{y_{2i}}\;\cdots {\;\pi }_{r-1}{\left(x_{i}\right)}^{y_{\left(r-1\right)i}}\right]\right]$$

where:

$\tilde{\beta }$ is the estimator value to maximize $L\left(\beta \right)$, we need the first and second derivates to function $L\left(\beta \right)$, and the β value is determined using the Newton-Rapson iteration method [54].

## 4. Results

Table 2 shows that −2 Log-likelihood for the final model was less than 80, the measure computed for all independent variables in multinomial logistic regression. The difference between these two measures was 772.231 for testing statistical significance. The test is like the F-test. This metric tests whether the improvement is associated with other variables. In other words, Table 2 shows the statistical metrics for evaluating the null model (intercept-only model) and the final model with the intercept and all independent variables. We found that the final model was statistically significant (Chi-Square of 772.231, *p* < 0.001). These results show that the final model was better than the intercept-only model.

The model explained more than 48% of the variability based on the Nagelkerke R-square with no evidence of poor fit, for example, Pearson Chi-Square = 10.227, *p* = 0.249, Deviance Chi-Square = 10.127, *p* = 0.256 (see Table 3 and Table 4).

Table 5 shows the statistically significant variables estimating the dependent variable. All these inputs such as economic status, residence, and car ownership in the household were statistically significant variables (*p* < 0.05). Moreover, we found that gender was not a statistically significant variable, with *p* > 0.05. However, this variable was excluded from the presented model.

The chi-square statistic is the difference in −2 Log-likelihoods between the final and reduced models. The reduced model was formed by omitting an effect from the final model. The null hypothesis claimed that all parameters of that effect are 0. The reduced model was equivalent to the final model because omitting the effect does not increase the degrees of freedom.

Table 6 reveals that almost 25 times more respondents living in a household without a car compared to the reference group (household with a car) most often walked to work or school rather than by car. In addition, the results demonstrate that almost 4% of respondents commuted to work or school from another city or village compared to the reference group by walking rather than by car. Finally, employees were less likely to walk to work than students to school when compared between walking and car. Second, the results show that more than 33 times more respondents living in a household without a car compared to the reference group use public transport to get to work or school more often than using a car. In addition, we found that employees also used public transport services to a lesser extent compared to the reference group representing students in the comparison of public transport versus the car. Finally, the multinomial logistic regression model showed that more than 16% of respondents compared to the reference group commuting to work or school from another city or countryside used public transport less than a car.

Table 7 shows that the multinomial logistic model correctly classified 67% of all passengers with an emphasis on the mode of transport for commuting to work or school as walking, public transport, and car based on three independent input variables: economic status, residence, and car ownership. The results show that the model best predicts walking and car as modes of transport, by more than 70%. However, less than 11% of pedestrians were estimated correctly by the multinomial logistic model. The main diagonal correctly explains the estimated mode of transport. Other values indicate that the model incorrectly classified the respondent. For example, 10 respondents commuting to school or work on foot were determined as respondents using public transport according to the model. In other words, these results indicate that these respondents walking to work, or school did not differ from respondents using public transport, based on the selected independent variables. This value indicates that future research can focus on expanding the current three statistically significant variables by at least one variable increasing the prediction of respondents commuting by public transport to work or school. One such variable could be the ownership of a pre-paid ticket or other variable. We further compared the overall classification with the chance accuracy rate to assess the relevance of the model.

The chance accuracy rate

(7) $$\left[{\left(0.243\right)}^{2}+{\left(0.197\right)}^{2}+{\left(0.560\right)}^{2}=0.411\right]$$

is calculated based on the proportion of cases for each group of the response variable. The model is helpful if the total classification rate is 1.25 times higher like chance accuracy [55].

(8) 1.25∗0.411=0.514∗100=51.40%

We find that the model accuracy was higher by more than 15% compared to the chance criterion. 

## 5. Discussion

We aimed to discover the specific behavior of passengers when commuting to work and school during the COVID-19 period in a sample of almost 2000 respondents from two EU countries. Significant factors influencing the choice of means of transport were statistically shown. We proposed three hypotheses reflecting the relationship between economic status, member households with a car, and the residence of respondents regarding commuting to work and school.

The issue of the impact of the pandemic on changing the mindset of society and related habits has been analyzed in many studies. The change in the perception of the population in the field of mobility was applied in many of them through multinomial logistic regression.

A study in South America reflecting the situation in the state of Chile used an online questionnaire for data collection. It focused on predicting the perception of society in the context of changes in the economy, due to the onset of the COVID-19 pandemic, using a multinomial logistic regression model for this purpose [56]. In the question of choosing an alternative mode of transport, research was conducted to predict determination of the choice of the most suitable infrastructure using a regression model. The need to have sufficient infrastructure for active transport was pointed out, while the connection between the preference and infrastructure separated from road vehicles was proven [57]. Another study analyzed the perception of the Spanish public during the COVID-19 period. It compared the level of risk for a group of workers from home and others who used transport to work. Using a logistic regression model, the level of risk perception was evaluated according to sociodemographic variables [58]. Assessment of risk perception during COVID-19 was also analyzed in Seattle. Drivers were investigated and their risk-related behaviors were evaluated through regression models [59]. A study investigated the risk perception and changing behavioral attitudes of passengers in rail transport in Indonesia. Dimensions of health, psychology, and time were assessed. Anxiety about using public transport during the pandemic period was confirmed [60]. The intention of commuters to choose rail transport and other modes of transport was evaluated with a logistic regression model according to personal attributes, travel attributes, and perception of COVID-19. The transition of respondents from rail transport to private car, walking, or bicycle transport was demonstrable [61]. Changing commuting behavior in the Netherlands was reflected from a survey using smartphone GPS signals. A logistic regression model was again used to assess increased telecommuting during COVID-19 and the use of car transport considering factors such as sociodemographic characteristics, initial travel behavior, and initial work situation. The results showed reduction in the use of cars and an increase in walking and bicycling [62]. Changes in travel behavior in the context of the COVID-19 pandemic in public transport were also analyzed in France. A survey analyzed the flow of public transport, passenger cars, and bicycles. A transition to teleworking caused a decrease in public transport. Active transport recorded an increase due to the transfer of passengers from public transport [63]. Travel mode choice was reflected in another study. Means of public transport were defined as a possible source of infection. Higher-income groups of residents travelled less by public transport. In our study, we came to a similar conclusion, that students use public transport more often, and employees use cars more [64]. A mobility survey among students and employees was also conducted in Italy at the University of Padova. Logistic regression models were used to predict the factors influencing travel decisions, considering travel habits, attitudes, and opinions [65]. Identification of walking behavior determinants was another topic that was analyzed in detail by the type of walking, including commuting, non-commuting and social walking during the COVID-19 period [66]. A study in Bangladesh examined the shift to active transport and the factors influencing this change. Variables included people’s income, risk perception, bicycle ownership, and infrastructure availability [67]. Factors affecting commuting were also analyzed in Philadelphia. The participants were divided into individual, social, and environmental groups to assess the impact on active transport [68]. The difference between the perception of risk between means of transport in shared modes and private modes owners was analyzed in this study. An increased perceived risk of contagion was confirmed with respect to public transport, as well as shared means compared to private modes of transport [69]. A change in urban mobility in Brussels due to restrictive pandemic measures was evaluated in another survey. An increase in cycling and a slower increase in urban traffic were found [70].

The EU presented a proposal for a sustainable development strategy to identify and develop measures to achieve continuous quality improvement. In the context of investigating the mode of transport in daily mobility, it is important to consider the idea of sustainable development. Many studies have been devoted to this paradigm with the direction of continuous advancement of transport provision [71,72,73]. The setting of an exact transport policy is a prerequisite for achieving mutual synergistic effects of all types of transport to achieve the principles of sustainable development.

In our study, a sample of mostly young respondents from two EU states was engaged using an online questionnaire monitoring traffic behavior during the COVID-19 period. This can be considered a limitation of the study. In further research, we would like to focus on expansion and diversification of countries, as well as a wider range of respondents. In future research, we would like to focus on the expanded understanding of travel behavior and the possibilities of supporting public transport and the connection with alternative modes of transport following the sustainable development initiative set by the EU.

## 6. Conclusions

The choice of transport means is influenced by many factors, and the impact of the onset of the COVID-19 pandemic was reflected in travel behavior. Our study was devoted to evaluating the preference for means of transport based on several variables.

We dealt with prediction of traffic behavior using a multinomial regression model during the COVID-19 pandemic. The selected prediction model estimated the most used modes of transport according to independent variables with an accuracy of almost 70%. Based on independent variables (including economic status, residence, and car ownership), means of transport for commuting to work and school were found. Respondents preferred the car, walking, and lastly, public transport, as the most often used means of transport. First, our study showed that almost 25 times more respondents living in a household without a car, compared to the reference group (household with a car), preferred walking to a car. The minority of these respondents in the analyzed group commuted to work or school from another city or village by walking. Second, more than 33 times respondents living in a household without a car compared to the reference group (household with a car) used public transport rather than a car. Public transport services were used by employees to a lesser extent than students. The results show that students mainly used public transport withing the city in which they had permanent residence.

Based on many studies and the results of our research, there has been an obvious decline in public transport preferences due to the COVID-19 pandemic. The purpose of our study was to identify traffic behavior in daily mobility for work or school as influenced by COVID-19 pandemic. Following the idea of sustainable development of the EU and in the setting of the transport policy, our research results point to future possible space for the development of public transport services, as well as alternative modes of transport. Our prediction model for estimating mode of transport would be a prerequisite for planning public transport activities in cooperation with passengers’ needs and requirements for ensuring mobility. Improving public transport services and promoting shared scooters and bicycles would lead to a reduction in carbon footprint and elimination of traffic jams during morning and afternoon rush hours.

Although limitations about the age of the respondents and the geographical sample were defined, the conducted research can be perceived as innovative. The research dealt with the prediction of passenger behavior and the choice of transport mode in daily mobility. The results could be useful for managers for the creation of transport policy, operators of transport services, as well as passengers themselves, to ensure mobility following the needs of the traveling public.

## Figures and Tables

**Table 1:** Case Processing Summary.

		N	Marginal Percentage
**How do you most often get to work or school?**	Walking	348	24.30%
	Public transport	283	19.70%
	Car	803	56.00%
**Economic status**	Employee	443	30.90%
	Student	991	69.10%
**Do you live in the city where you work or study?**	No	691	48.20%
	Yes	743	51.80%
**Are you a household member with a car?**	No	265	18.50%
	Yes	1169	81.50%
**Valid**		1434	100.00%
**Missing**		558	
**Total**		1992	
**Subpopulation**		8	

**Table 2:** Model Fitting Information.

Model	Model Fitting Criteria			Likelihood Ratio Tests		
	AIC	BIC	−2 Log Likelihood	Chi-Square	df	Sig.
**Intercept Only**	852.658	863.195	848.658			
**Final**	92.427	134.573	76.427	772.231	6	0.000

**Table 3:** Goodness-of-Fit.

	Chi-Square	df	Sig.
**Pearson**	10.227	8	0.249
**Deviance**	10.127	8	0.256

**Table 4:** Pseudo R-Square.

**Cox and Snell**	**0.416**
**Nagelkerke**	0.483
**McFadden**	0.272

**Table 5:** Likelihood Ratio Tests.

Effect	Model Fitting Criteria			Likelihood Ratio Tests		
	AIC of Reduced Model	BIC of Reduced Model	−2 Log Likelihood of Reduced Model	Chi-Square	df	Sig.
**Intercept**	92.427	134.573	76.427 ^a^	0.000	0	
**Economic status**	117.871	149.481	105.871	29.444	2	0.000
**Do you live in the city where you work or study?**	475.987	507.596	463.987	387.559	2	0.000
**Are you a household member with a car?**	330.704	362.313	318.704	242.277	2	0.000

Chi-square is the difference in −2 Log-likelihoods between the final model and a reduced model The reduced model was formed by omitting an effect from the final model. The null hypotheses was that all parameters of that effect are 0. ^a^ This reduced model is equivalent to the final model because omitting the effect does not increase the degrees of freedom.

**Table 6:** Parameter Estimates.

Mode of Transport^a^	Variable	B	Std. Error	Wald	df	Sig.	Exp(B)	95% C. I. for Exp(B)	
								Lower Bound	Upper Bound
**Walking**	Intercept	0.231	0.122	3.607	1	0.058			
	Economic status = employee = 0	−0.846	0.177	22.778	1	0.000	0.429	0.303	0.607
	Economic status = student = 1	0 ^b^			0				
	Do you live in the city where you work or study? = no = 0	−3.351	0.22	231.693	1	0.000	0.035	0.023	0.054
	Do you live in the city where you work or study? = yes = 1	0 ^b^			0				
	Are you a household member with a car? = no = 0	3.214	0.312	106.059	1	0.000	24.881	13.496	45.87
	Are you a household member with a car? = yes = 1	0 ^b^			0				
**Public transport**	Intercept	−0.377	0.136	7.678	1	0.006			
	Economic status = employee = 0	−0.786	0.185	17.945	1	0.000	0.456	0.317	0.656
	Economic status = student = 1	0 ^b^			0				
	Do you live in the city where you work or study? = no = 0	−1.808	0.176	105.467	1	0.000	0.164	0.116	0.232
	Do you live in the city where you work or study? = yes = 1	0 ^b^			0				
	Are you a household member with a car? = no = 0	3.498	0.301	135.491	1	0.000	33.061	18.344	59.586
	Are you a household member with a car? = yes = 1	0 ^b^			0				

^a^ The reference category is the car; ^b^ This parameter is set to zero because it is redundant.

**Table 7:** Classification.

Observed	Predicted			
	Walking	Public Transport	Car	Percent Correct
**Walking**	251	10	87	72.10%
**Public transport**	167	30	86	10.60%
**Car**	112	11	680	84.70%
**Overall percentage**	37.00%	3.60%	59.50%	67.00%

## Data Availability

All data used here are available on request from authors.
